# Routine testing of fetal Rhesus D status in Rhesus D negative women using cell-free fetal DNA: an investigation into the preferences and information needs of women

**DOI:** 10.1002/pd.4135

**Published:** 2013-06-20

**Authors:** Kerry Oxenford, Caroline Silcock, Melissa Hill, Lyn Chitty

**Affiliations:** 1 Fetal Medicine Unit, University College London Hospitals NHS Foundation TrustLondon, UK; 2 Women's Health Division, University College London Hospitals NHS Foundation TrustLondon, UK; 3 Clinical and Molecular Genetics, Great Ormond Street Hospital for Children NHS Foundation TrustLondon, UK; 4 Clinical and Molecular Genetics Unit, UCL Institute of Child HealthLondon, UK

## Abstract

**Objective:**

The goal of this study is to investigate women's preferences and information needs for routine implementation of fetal Rhesus D (RhD) typing using cell-free fetal DNA.

**Methods:**

A questionnaire was developed following focus groups and interviews with both health professionals and RhD negative (RhD−) women offered fetal RhD genotyping within a research study and distributed to RhD− women attending routine antenatal appointments in four National Health Service hospitals. Current knowledge of blood types, anti-D administration, fetal RhD genotyping and future practices were explored.

**Results:**

A total of 19 respondents participated in interviews and focus groups, and 270 respondents completed the questionnaires. Questionnaire respondents overwhelmingly felt that the test should be offered to all RhD− women (92.1%), and 75.9% said that they would accept this test. Most were happy to have the test even if it involved extra blood tests (89.3%) or appointments (79%). The knowledge of blood groups was poor. Although 90.7% knew that the baby could have a different blood group from themselves, only 34% knew that blood groups are inherited from both parents. More than 40% were not aware that anti-D would not be required if their baby was RhD−.

**Conclusions:**

Women would welcome the introduction of routine fetal RhD genotyping. Information leaflets and training of midwives will be essential for implementation to ensure good understanding regarding testing. © 2013 The Authors. *Prenatal Diagnosis* published by John Wiley & Sons Ltd.

## INTRODUCTION

To prevent alloimmunisation of Rhesus negative (RhD−) mothers carrying a Rhesus positive (RhD+) fetus, the National Institute for Health and Clinical Excellence recommends that routine antenatal prophylaxis with anti-D immunoglobulin should be offered to all RhD− pregnant women in the third trimester as well as after birth and following events associated with fetal maternal haemmorhage.^1^ As a result, the incidence of haemolytic disease of the newborn caused by alloimmunisation has fallen dramatically. However, in the UK, about 40% of RhD− women (around 40 000 per year) carry an RhD− fetus and thus receive anti-D unnecessarily.^2^ Anti-D is produced from pooled plasma from large numbers of RhD− donors who have been transfused with RhD+ red cells to stimulate the production of RhD antibodies^3^ and thus carries a very small risk of transmission of human blood-borne viral or prion diseases.^4^

The identification of cell-free fetal DNA (cffDNA) in maternal blood from early in pregnancy^5^ has allowed the development of non-invasive prenatal testing (NIPT) to determine the fetal RhD genotype in RhD− mothers by analysing a maternal plasma sample.^6^ This test has been used clinically in England for over a decade to direct care for sensitised RhD− women who would require additional monitoring and potential treatment if they were carrying an RhD+ fetus.^6^ Advances in technology mean testing can now be carried out accurately and efficiently on a larger scale using automated techniques.^7^,^8^ Indeed, routine testing at 25 weeks gestation has already been successfully introduced into antenatal care in Denmark.^9^

Recent guidance from the National Institute for Health and Clinical Excellence has recommended the exploration of routine antenatal fetal RhD genotyping.^10^ Here, we investigate how women view current information about blood groups, anti-D administration, the new cffDNA test and how they would like it offered in practice. This study forms part of a larger study developing standards for the implementation of routine fetal RhD genotyping in the UK (antenatal determination of fetal RhD status using cffDNA in the maternal circulation before 20 weeks gestation: is routine application practical and beneficial? PB-PG-0107-12005).

## METHODS

### Focus groups and interviews

To develop questionnaires for the main study, we used focus groups and one-to-one interviews to explore the views and experiences of RhD− women and health professionals at one London hospital. A purposive sampling method was used for recruitment. The RhD− women were those who had been previously offered with fetal RhD genotyping as part of an ongoing intervention study. The study invitation and information sheet were provided at the 28-week appointment, and participants were interviewed by the lead researcher (KO). Health professionals were identified from staff lists, invited in person and interviewed by one of two researchers (KO and CC) or took part in one of two focus groups. The study was approved by the National Research Ethics Committee London Bentham (07/H0714/128).

A semi-structured discussion guide was used to ascertain perceptions of the current antenatal information regarding RhD and anti-D administration for RhD− women, explore views and opinions regarding routine fetal RhD genotyping and identify preferences for implementation into routine practice (see online Appendix 1). The interviews and focus groups were recorded, transcribed verbatim and analysed using thematic analysis.^11^ To ensure inter-rater reliability, the transcripts were read and coded independently by two other researchers (MH, CC) with themes identified and agreed collectively. Data collection ceased when no new codes were identified.

### Questionnaire study

#### Design

The questionnaire was developed using significant themes identified from the focus groups and interviews. Questions included views and preferences regarding fetal RhD typing (*n* = 4), current knowledge of blood group, anti-D and its administration (*n* = 15), current sources of information (*n* = 8) and demographics and characteristics of the participants (*n* = 9). A four-item Likert scale was used for five of the questions to assess understanding of current information and knowledge and beliefs. A paragraph describing routine fetal RhD genotyping was given in the questionnaire (online supplementary data, S1). The questionnaire was initially piloted on 20 women, and no changes were needed.

#### Data collection

The questionnaire was distributed in four National Health Service hospitals, one London teaching hospital and three regional hospitals. Research midwives at each site invited RhD− women to fill in the questionnaire whilst waiting for a routine antenatal appointment any time after 12 weeks gestation.

#### Data analysis

Data were entered onto an Excel spreadsheet and analysed using spss statistics version 17.0. Descriptive statistics were used to analyse individual questions, which included the 15 questions testing the knowledge of blood group, anti-D and the reason why anti-D is given. One point was given for each correct answer and totalled to give an overall knowledge score ranging between 0 and 15. A one-way analysis of variance was used to test for differences in knowledge scores compared with several variables. A *t*-test was used to test if knowledge scores varied in the second and third trimester. Chi-square tests were carried out to compare answers to specific questions in the second and third trimester.

Spearman correlation was used to determine whether there was a relationship between knowledge scores for anti-D and blood group compared with the perception of how useful the information was, the level of information provided and how knowledgeable participants believed themselves to be.

Women were allowed to give more than one response for the questions relating to how they receive information and reasons for accepting or declining NIPT. For these questions, descriptive analysis was used with percentages being calculated from the total number of responses. One question asked women to rank their answers in order of importance. Some women only ranked those they rated as most important, and these responses were included. This question was analysed by taking each option individually and calculating how many people had ranked it at each level.

## RESULTS

### Qualitative results

#### Participants

Six women and 13 health professionals recruited from one London hospital participated. Six one-to-one interviews were held with RhD− women, two with obstetric registrars and two with midwives. Two focus groups were held with midwives (*n* = 9). The women represented a variety of experiences with regard to receiving different fetal RhD genotyping results and decisions regarding anti-D (Table 1). Although only six women were included, this was sufficient to reach a point where no new themes were identified.

**Table 1:** Interview participants: Rhesus negative women

Name	Parity	Predicted RhD status using NIPT	Anti-D
Woman 1	0	Positive	Yes
Woman 2	0	Negative	Yes
Woman 3	0	Positive	Yes
Woman 4	0	Negative	Declined
Woman 5	0	Positive	Yes
Woman 6	2	Negative	Declined

RhD, Rhesus D factor; NIPT, non-invasive prenatal testing.

### Perceptions of routine fetal genotyping

Several themes emerged regarding perception of the new test and how it should be offered into practice.

#### Benefits of fetal RhD genotyping

All participants felt that the new test was a positive development that should be offered to all RhD− women (Table 2; quotes 1 and 2). Health professionals felt that, overall, women were not concerned about anti-D being a blood product. Women considered the benefits of anti-D outweighed any risks (Table 2; quote 3) but were keen to avoid unnecessary treatment (Table 2; quotes 4 and 5).

**Table 2:** Quotes from participants of interviews and focus groups

Quote number	Participant	Quote
1	RhD− woman	‘…Oh I think it would be great absolutely and I would be all for it.’
2	Midwife	‘…they are very excited that you can find out the blood group of the baby, before the babies born with no invasive procedure.’
3	RhD− woman	‘I am aware of that risk and I think the benefits outweigh the risk.’
4	RhD− woman	‘..you know if you don't have to have a blood product I don't see why you should be given it.’
5	RhD− woman	‘..Yes it is a blood product, and I think ideally you know if you can avoid having blood products that's great…’
6	Midwife	‘… when you have so many bookings squished into a clinic and you have all these documents and all these things to go through.’
7	Midwife	‘I think it will change practice positively providing that you've got a good accuracy rate.’
8	RhD− woman	Blood tests….‘I think there was an extra one I'm not sure, but that doesn't bother me…’
9	RhD− woman	‘I think if it becomes routine that they know they have to go and have the blood test then they will just accept it.’
10	RhD− woman	‘I guess pretty close to 100% accurate, otherwise then if you don't have the injection you are risking the health of the child aren't you’
11	RhD− woman	‘I'm not saying I haven't been given all the information but there is just too much to take in during pregnancy’
12	Midwife	‘…so I try to make it more graphical then because its difficult to explain what its all about.’
13	RhD− woman	‘…the booklet one was really good, that kind of taught me because I didn't know about it before …it's still a bit mind boggling but easier when its in a diagram.’
14	Midwife	‘I think when the information leaflets were being sent to them then it made our job easier. Because they would have read about the information and at least they are well informed.’
15	RhD− woman	‘I think it's always nice to have something written down so you can refer to it after if you don't take it all in at the time.’

RhD−, Rhesus D negative.

#### Service delivery

Health professionals felt that although offering the test early in pregnancy was important, offering additional appointments was not practical because of staffing levels and time required. Concern was raised that time constraints would make it difficult to discuss at booking (Table 2; quote 6). The routine 16-week appointment was suggested as a good opportunity to offer the test to RhD− women. However, most women were happy to have a separate blood test and attend for an extra appointment if necessary (Table 2; quote 9).

#### Test accuracy

All participants thought that accuracy of the test was highly important (Table 2; quotes 11 and 12) and a key factor in the acceptance of the test. Women want to be offered the test at a time when it is most accurate even if this meant it was later in pregnancy. Two women felt that they would still rather have anti-D ‘to be on the safe side’; however, these women said that if the test was proven to be accurate and offered as part of routine care rather than on a research basis, then they would feel confident about not having anti-D. Health professionals were concerned about false negative results and the potential for babies developing preventable haemolytic disease of the newborn.

#### Information and education

Information and education were also major factors highlighted by all participants. Women felt that the amount of information given in pregnancy can be difficult to absorb, and although they said that they did not always read it, they still felt that receiving simple and succinct written information to the appointment where the test is offered was important (Table 2; quotes 13, 16, and 17). Diagrammatic information was generally preferred (Table 2; quotes 14 and 15).

Midwives recognised that they would be the primary source of information about the test and wanted extra training, written and face-to-face. Notably, health professionals felt that most women accept whatever is offered to them as routine care (Table 2; quote 10), an observation confirmed by women who stated that they would accept their recommendations (Table 2, quote 8) as they trusted health professionals to do what was best for women.

### Quantitative results

#### Participants

A total of 270 of the 287 women approached completed the questionnaire (response rate: 94%), which were developed using the themes emerging from the qualitative study. Four questionnaires were excluded because the participant was under 12 weeks gestation. Demographic information of participants is available as supplementary data (S3).

#### Current knowledge about Rhesus D status and anti-D

The average knowledge score (on a scale of 0–15) was 8.7 (SD = 3.44, range = 0–15): responses are shown in Table 3. Knowledge score comparisons with several variables are shown online in Table S3. There was a significant association between education and knowledge score [*F* (5, 240) = 7.956, *p* < 0.001]. Post *hoc* tests showed that women with a degree [*M* = 10.20, 95% CI (9.54, 10.85)] scored significantly higher than women with either no qualifications [*M* = 6.53, 95% CI (5.51, 7.56)] *p* = 0.001 or educated to general certificate of secondary education level [*M* = 7.43, 95% CI (6.63, 8.22)], *p* < 0.001.

**Table 3:** Fifteen questions testing current knowledge of blood group and anti-D

Knowledge questions	Correct	Wrong	Unsure
1) I inherited my blood group from both parents	33.7% (91)	33.3% (90)	32.2% (87)
2) I will be offered an anti D injection in pregnancy	90.0% (243)	3.3% (9)	6.3% (17)
3) My baby could have a different blood group from me	90.4% (243)	1.1% (3)	8.1% (22)
4) Rhesus negative means you do not have the D antigen on your red blood cells.	38.1% (103)	5.9% (16)	55.6% (150)
5) Is anti D a blood product?	41.9% (113)	33.7% (91)	23% (62)
6) Can anti D cause an allergic reaction	32.6% (88)	7% (19)	57.4% (155)
7) Anti D is made from human blood plasma	27.4% (74)	4.1% (11)	66.3% (179)
8) Anti D is given by injection	91.1% (246)	0.4% (1)	6.7% (18)
9) Anti D is strictly controlled to avoid transmission of blood borne infections	51.1% (138)	3.3% (9)	43.7% (118)
10) If you are RhD− and your baby is RhD− would you need Anti D	55.9% (151)	17.4% (47)	24.4% (66)
11) Anti D is given to prevent the body producing antibodies	68.9% (186)	7% (19)	21.9% (59)
12) Anti D is given to protect babies in future pregnancies	69.3% (187)	9.3% (25)	19.3% (52)
13) Anti D is given because the baby might be Rhesus positive	68.5% (185)	4.1% (11)	24.4% (66)
14) Anti D is given because the baby might be Rhesus negative	59.6% (161)	8.1% (22)	27.4% (74)
15) After my baby is born, I will be given anti D if my baby has a positive blood group	48.9% (132)	26.3% (71)	23% (62)

a Values reported as % (*n*). Some responses were missing therefore total values may not add up to 100%.

There were significant differences in knowledge score in relation to whether women had anti-D in their pregnancy [*F* (2, 255) = 13.027, *p* < 0.001]. Post *hoc* comparisons showed that women who were unsure if they had received anti-D (*M* = 4.42, SD = 3.605) scored significantly lower than both women who knew they had received anti-D (*M* = 9.26, SD = 3.160) *p* < 0.001 and those that knew they had not received it (*M* = 8.45 *SD* = 3.290).

When questions were analysed individually, women in their third trimester were more likely to know that they would not need anti-D if they knew their baby was RhD− [65.6% vs 48.1% (*p* < 0.005)] and that they would only be given anti-D after birth if their baby was RhD+ [59.8% vs 39.7% (*p* < 0.005)]. Results showing variations across trimesters are shown online in Table S4.

#### Current sources of information

The main source of information for women was their midwife (50.3%), followed by the hospital information leaflet (17.7%), Internet (10.8%), family and friends (9%), doctor (7.5%) and other sources including books and prior knowledge through education or previous pregnancies (4%). Three women said they had not received any information regarding their blood group.

The majority of women (68.50%) felt that they had received enough information about their blood group and had found it useful (80.7%). Half of the women (50.6%) felt they knew ‘a little bit’, and 31.8% felt they were ‘quite’ knowledgeable about their blood group. The majority of women (80.1%) had been told about anti-D in their current pregnancy. Significantly, more women in their third trimester had been told about anti-D compared with those in their second trimester (86.5% vs 74.2%, *p* < 0.05). The majority of women felt they had been given enough information about anti-D (67.7%). However, a significant number felt they needed more information (32.3%). Women were more likely to feel that they had been given enough information if they were in their third trimester (76.3% vs 59.4% *p* < 0.005). Generally, women found the information that was provided about anti-D useful (79.2%).

#### Opinions on routine fetal RhD genotyping

Women were overwhelmingly in favour of the test being offered routinely to all women (92.1%). However, there was some uncertainty when asked if they would accept the test themselves (75.9%). The possible reasons for declining the test are given in Table 4.

**Table 4:** Views of potential routine fetal Rhesus D typing

Total (*n* = 270)^a^	No	Yes	Unsure
Should fetal RhD typing be offered to all RhD− women? (*n* = 215)	0.9% (2)	92.1% (198)	7% (15)
Would you accept the test? (*n* = 216)	2.8% (6)	75.9% (164)	21.3% (46)
Would you need further information about the test? (*n* = 267)	47.2% (126)	36.7% (98)	16.1% (43)
How do you prefer to receive information?^b^			
Midwife	59.8% (104)		
Hospital information leaflet	34.5% (60)		
Internet	4.6% (8)		
Other	1.1% (2)		
When would you want to receive information?^b^			
In post with booking letter	23.1% (31)		
At booking appointment with midwife	46.3% (62)		
In the post with blood group results	21.6% (29)		
On the day of NIPT	6.7% (9)		
Other	2.2% (3)		
Why would you want the test?^b^			
Rather avoid anti-D	32.7% (88)		
Rather avoid injection	22.3% (60)		
To know more about the baby as possible	28.6% (77)		
If recommended by midwife	16.4% (44)		
Why would you not want the test? b			
Would not want extra blood test	20.8% (5)		
Would want anti-D to be on the safe side	37.5% (9)		
Would want more information	41.7% (10)		

a Total number responses shown for individual questions (*n*).

b Respondents could select more than one answer, % have therefore been calculated from the total number of responses for each answer.

RhD, Rhesus D; NIPT, non-invasive prenatal testing.

There was a significant difference in the knowledge score for women who were unsure if they would accept the test and those that would accept the test (*p* = 0.002). Women who were unsure if they would accept fetal RhD genotyping had significantly lower knowledge scores [*M* = 7.64, 95% CI (6.66, 8.63)] than those who would accept the new test [M = 9.49, 95% CI (8.99, 9.99)], *p* = 0.002. Other comparisons and demographic characteristics were not significant.

#### Preferences for how fetal RhD genotyping should be offered

**Figure 1:**
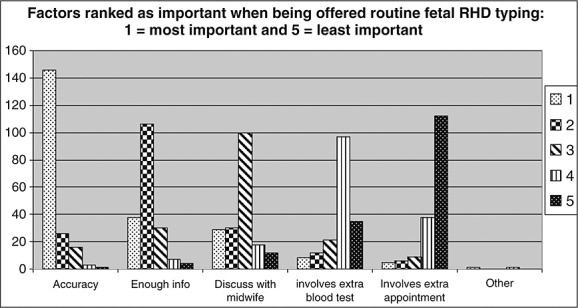
Factors ranked as important when being offered routine fetal RhD typing

The majority of women (95.9%) would rather have the blood test performed at the same time as other routine blood tests. However, they would be happy to have an extra blood test (89.3%) if it was necessary. Most women would want the opportunity to discuss the test with a midwife (89%) and would be willing to have an extra appointment if required (79%). Women ranked the test accuracy, having enough information and being able to discuss with a midwife most highly (Figure 1).

#### Information preferences

Most women want to receive information from their midwife (59.7%) or have written information in a leaflet (34.5%) rather than accessing the information on the hospital website (4.6%). Most wanted to be told about the test at the booking appointment with the midwife (46.3%), with 23.1% preferring to receive information in the post with the initial booking appointment letter and 21.6% wanting it posted with the results of their blood test. There was a clear preference for receiving the information prior to the day of the test (91%).

Just under half the women (47.2%) said that the amount of information provided in the study leaflet was sufficient, 36.7% wanted more information and 16.1% were unsure. The following topics were identified as additional information that would be useful:

1. Risks or side-effects to mother and baby
2. Timing of tests, if extra appointment needed
3. Implications of results
4. How the test works
5. Accuracy and implications if the test result is incorrect
6. Being able to discuss the test with a midwife/health professional
7. General information about blood group, why the test is necessary, risks of anti-D
8. Whether any other information can be found out from the test.

### DISCUSSION

In this unique study, we have clearly demonstrated that there is enthusiasm from both women and health professionals for routine fetal RhD genotyping using cffDNA in maternal blood, regardless of whether it will require extra blood tests or visits. However, health professionals felt that it should be offered with other routine appointments to minimise resource implications, an observation that is supported by the economic evaluation performed in this study.^12^

The strength of the study is the use of qualitative data from women with experience of fetal RhD genotyping and health professionals to develop the questionnaire together with the quantitative data on preferences from a large number of RhD− women attending four different hospitals. This work expands the current research on fetal RhD genotyping which to date has focused on technology development, test accuracy and economics.^6^–^8^ Previous research into views and experiences of the use of cffDNA tests is based on tests already in clinical practice and includes women^13^,^14^ and health professionals'^15^ views on fetal sex determination for clinical indications. Other studies have looked at public,^16^ pregnant women^17^,^18^ and health professionals'^19^ views of NIPT using cffDNA for Down's syndrome and other conditions. These studies found that NIPT is viewed as a positive step in prenatal diagnosis for Down's syndrome and genetic conditions.

This study found that women would be more likely to accept fetal RhD genotyping if they felt confident about its accuracy. Women with lower knowledge scores were less certain whether they would accept the test. It is concerning that knowledge scores showed a lack of understanding of current care regarding anti-D in several areas. Women were unsure why anti-D is given and that their baby could have a different blood group from them. This knowledge improves in the third trimester, most likely because information is given at the time of anti-D administration at the beginning of the third trimester. Women wanted to know why routine RhD genotyping is beneficial, and thus understanding blood group inheritance and why anti-D prophylaxis is only necessary if the baby is RhD+ is important.

Women showed a preference for receiving written information in the post and speaking to the midwife before being offered the test. This reflects findings from a Dutch study evaluating screening programmes for non-Rh red blood cells; when asked about their information needs, women desired written information and prioritised having information on the clinical implications for both mother and child.^20^ One of the key issues women wanted addressed was whether fetal RhD genotyping testing posed a risk to mother or baby. The test was described as a ‘normal blood test’. However, women were still concerned about risk. Furthermore, as we are using the baby's DNA, there was also concern that we could potentially reveal other information about the baby.

A notable theme from the qualitative data was women being willing to accept whatever was recommended to them by the midwife or presented as routine care. Questionnaire findings showed 16.7% of women felt that they would accept the test if it was recommended to them by the midwife. This is a similar finding to a study that investigated women's interest and expected uptake of NIPT using cffDNA for prenatal diagnosis, which found that 20% of respondents would do what their doctor recommended.^17^

These findings indicate that health professionals, in particular midwives, will need to ensure that complete and balanced information is given to women to allow them to make an informed decision regarding fetal RhD genotyping and anti-D administration. Current blood group information leaflets could be developed to include information about fetal RhD genotyping. Information should be clear and concise and presented at a relevant time. This study cannot determine when in the care pathway this test should be offered, but inherently, the earlier in pregnancy, the better to avoid unnecessary anti-D administration for early sensitising events. As information regarding the mother's own Rh type is needed before fetal RhD genotyping can be interpreted, sending the information with the booking blood results seems appropriate and is a time when there is less information being given about other aspects of pregnancy.

#### Study limitations

In this study, we attempted to gather the views and preferences of a cross-section of women by recruiting from four different hospitals in different regions of the UK. However, several issues may limit how representative our findings are. For example, the majority of pregnant women who took part in this study were White, and women from ethnic minority groups are under-represented.

A small sample of health professionals and RhD− women took part in the focus groups and interviews: These were all based at one hospital and only had experience of fetal RhD genotyping being offered on a research basis. Other hospitals may have different protocols, and fetal RhD genotyping may be offered differently when introduced routinely into practice. Therefore, their views and experiences may be specific to their own experience. The study reflects women's stated preferences and may not in fact reflect future attitudes and uptake of fetal RhD genotyping. It will be important to research the best ways to provide information and education for women.

### CONCLUSIONS

This is the first study to investigate health professionals and women's views and opinions of routine fetal RhD genotyping. Although we have shown an overwhelmingly positive response, we have also demonstrated significant weakness in delivery of information regarding the current information on Rh blood groups and anti-D administration. Before being offered this new test, women want timely information on its benefits, risks, accuracy and implications, making development of information leaflets and health professional training key to routine clinical implementation. This work is critical for the development of policies and guidelines for the introduction of fetal RhD genotyping into routine care.

## Supporting Information

Funding sources: The National Institute for Health Research (NIHR) for Patient Benefit programme (PB-PG-0107-12005), the Central and East London NIHR Comprehensive Local Research Network and the Northumberland, Tyne and Wear NIHR Comprehensive Local Research Network funded the research. Professor Lyn Chitty is partially funded by the Great Ormond Street Hospital Children's Charity and by NIHR Biomedical Research Centre funding. The research fund is independent, and the views expressed in the paper are those of the authors and not necessarily those of the NHS, the NIHR or the Department of Health. Conflicts of interest: None declared

## References

[1] National Institute for Clinical Excellence (NICE) (2002). Technology appraisal guidance 41. Guidance on the use of routine antenatal anti-D prophylaxis for RhD-negative women.

[2] Daniels G (2002). Human Blood Groups.

[3] Kumpel BM (2002). Monoclonal anti-D development programme. Transpl Immunol.

[4] Kenny-Walsh E (1999). Clinical outcomes after hepatitis C infection from contaminated anti-D immune globulin. Irish hepatology research group. N Engl J Med.

[5] Lo YM, Corbetta N, Chamberlain PF (1997). Presence of fetal DNA in maternal plasma and serum. Lancet.

[6] Daniels G, Finning K, Martin P, Massey E (2009). Non-invasive prenatal diagnosis of fetal blood group phenotypes: current practice and future prospects. Prenat Diagn.

[7] Finning K, Martin P, Summers J (2008). Effect of high through put RHD typing of fetal DNA in maternal plasma on use of anti-RhD immunoglobulin in RhD negative pregnant women: prospective feasibility study. BMJ.

[8] Chitty LS, Finning K, Massey E (2011). Antenatal determination of fetal Rhesus (RH) D status using cell-free fetal DNA in the maternal circulation before 20 weeks gestation: is routine application practical and beneficial?. Arch Dis Child Fetal Neonatal Ed.

[9] Clausen FB, Christiansen M, Steffensen R (2012). Report of the first nationally implemented clinical routine screening for fetal RHD in D- pregnant women to ascertain the requirement for antenatal RhD prophylaxis. Transfusion.

[10] National Institute for Clinical Excellence (NICE) (2008). Technology Appraisal Guidance 156. Routine antenatal anti-D prophylaxis for women who are Rhesus D negative.

[11] Braun V, Clarke V (2006). Using thematic analysis in psychology. Qual Res Psychol.

[12] Chitty LS, Finning K, Wade A (2012). Routine fetal RHD typing using cffDNA in RhD negative women: timing, costs and efficiency. Prenat Diagn.

[13] Lewis C, Hill M, Skirton H, Chitty L (2012). Non-invasive prenatal diagnosis for fetal sexing – what is the value for service users?. Eur J Hum Genet.

[14] Lewis C, Hill M, Skirton H, Chitty L (2012). Fetal sex determination using free fetal DNA: service users' experiences and preferences for how the service should be offered in clinical practice. Prenat Diagn.

[15] Hill M, Compton C, Lewis C (2012). Determination of fetal sex in pregnancies at risk of haemophilia: a qualitative study exploring the clinical practices and attitudes of health professionals in the United Kingdom. Haemophilia.

[16] Kelly SE, Farrimond HR (2012). Non-invasive prenatal genetic testing: a study of public attitudes. Public Health Genomics.

[17] Tischler R, Hudgins L, Blumenfeld YJ (2011). Non-invasive prenatal diagnosis: pregnant women's interest and expected uptake. Prenat Diagn.

[18] Kooij L, Tymstra T, Berg P (2009). The attitude of women toward current and future possibilities of diagnostic testing in maternal blood using fetal DNA. Prenat Diagn.

[19] Sayres LC, Allyse M, Norton ME, Cho MK (2011). Cell-free fetal DNA testing: a pilot study of obstetric healthcare provider attitudes toward clinical implementation. Prenat Diagn.

[20] Koelewijn JM, Vrijkotte TG, de Haas M (2008). Women's attitudes towards prenatal screening for red blood cell antibodies, other than RhD. BMC Pregnancy Childbirth.

