# Supplementary material for: Routine testing of fetal Rhesus D status in Rhesus D negative women using cell-free fetal DNA: an investigation into the preferences and information needs of women
Source: Prenat Diagn. 2013 Jun 20;33(7):688–94. doi: 10.1002/pd.4135 (PMC4286012; doi:10.1002/pd.4135)
Supplement: Supplementary file 1 — Supporting Information [file pd0033-0688-SD1.doc]

**Online supplementary data**

(S1) Paragraph given in the Questionnaire explaining fetal *RHD* genotyping:

A new blood test has been developed. We can identify the baby’s DNA, which contains his / her genes circulating in your blood. By taking an ordinary blood sample from you we can use this genetic material to determine your baby’s blood group and so determine whether or not you need anti-D.

**Table S2: Demographics of questionnaire study participants.**

|  | **Total (n = 270)^a^** |
| --- | --- |
| **Age (years): (n=250)** |  |
| 15 – 20 | 9.2% (23) |
| 21 – 30 | 44.4% (111) |
| 31 – 40 | 42% (105) |
| 41 – 50 | 4.4% (11) |
| **Gravida: (n=269)** |  |
| 0 | 36.1% (97) |
| 1 | 34.2% (92) |
| 2 + | 29.7% (80) |
| **Number of children: (n=270)** |  |
| 0 | 46.3% (125) |
| 1 | 32.2% (87) |
| 2 + | 21.5% (58) |
| **Trimester: (n=268)** |  |
| 2^nd^ (12 – 27) | 50% (134) |
| 3^rd^ (28 – 42) | 50% (134) |
| **Ethnicity: (n=251)** |  |
| Asian or Asian British | 1.6% (4) |
| Black or Black British | 2.4% (6) |
| Mixed | 2.0% (5) |
| White | 92.8% (233) |
| Other ethnic group | 1.2% (3) |
| **Education: (n=247)** |  |
| No qualifications | 6.5% (16) |
| A level or equivalent | 17.8% (44) |
| Teaching or other non degree | 2.8% (7) |
| GCSE or equivalent | 27.8% (75) |
| Degree or equivalent | 30.4% (81) |
| Other qualification | 5.3% (13) |
| Post graduate degree | 4.5% (11) |
| **Religion: (n=242)** |  |
| None | 66.5 % (161) |
| Catholic | 9.9% (24) |
| Christian | 11.6% (28) |
| Church of England | 5.8% (14) |
| Jewish | 1.2% (3) |
| Russian Orthodox | .4% (1) |
| Muslim | 2.9% (7) |
| Protestant | .8% (2) |
| Sikh | .4% (1) |
| Methodist | .4% (1) |

^a^ Total number responses shown for individual questions (n)

Table S3: Knowledge score comparisons across variables

|  | **(n)** | **Knowledge score mean (SD)** | **p. value** |
| --- | --- | --- | --- |
| **Education Level** |  |  | <.001 |
| No qualification | 15 | 6.5 (1.8) |  |
| GCSE | 75 | 7.4 (3.5) |  |
| A level | 44 | 9.1 (3.3) |  |
| Degree | 81 | 10.2 (3) |  |
| Post graduate | 11 | 10.0 (3.3) |  |
| Other | 20 | 8.1 (3.4) |  |
| **Age group** |  |  | .004 |
| 15 – 20 | 23 | 7.4 (3.6) |  |
| 21 -30 | 111 | 8.1 (3.5) |  |
| 31 – 40 | 104 | 9.6 (3.2) |  |
| 41 - 50 | 11 | 8.1 (3.3) |  |
| **Ethnicity** |  |  | .774 |
| Asian or Asian British | 4 | 7.5 (5.2) |  |
| Black or Black British | 6 | 8.7 (1.7) |  |
| Mixed | 5 | 10.4 (4) |  |
| White | 232 | 8.7 (3.5) |  |
| Other | 3 | 9.3 (2.9) |  |
| **Parity** |  |  | .321 |
| No children | 124 | 8.6 (3.6) |  |
| 1 child | 87 | 9.1 (3.3) |  |
| 2 or more children | 58 | 8.2 (3.3) |  |
| **Trimester** |  |  | <.001 |
| Second trimester | 133 | 7.95 (3.615) |  |
| Third trimester | 134 | 9.45 (3.104) |  |
| **Had Anti D in pregnancy** |  |  | <.001 |
| No | 102 | 8.45 (3.290) |  |
| Yes | 144 | 9.26 (3.160) |  |
| Unsure | 12 | 4.42 (3.605) |  |
| **Accept NIPT if offered** |  |  | .003 |
| No | 6 | 8.33 (2.422) |  |
| yes | 164 | 9.49 (3.236) |  |
| Unsure | 45 | 7.64 (3.290) |  |

Table S4: Comparisons between trimesters

|  | 2^nd^ trimester | | 3^rd^ trimester | |  |
| --- | --- | --- | --- | --- | --- |
|  | (n) | % | (n) | % | p. value |
| **Enough information about Anti D in pregnancy?** |  |  |  |  | .003 |
| Agree | 79 | 59.4 | 100 | 76.3 |  |
| Disagree | 54 | 40.6 | 31 | 23.7 |  |
|  |  |  |  |  |  |
| **Been told about Anti D in pregnancy?** |  |  |  |  | .012 |
| Yes | 98 | 74.2 | 115 | 86.5 |  |
| No or unsure | 34 | 25.8 | 18 | 13.5 |  |
|  |  |  |  |  |  |
| **Need Anti D if baby we knew the baby had Rhd- blood group?** |  |  |  |  | .013 |
| Yes | 30 | 22.9 | 17 | 13.0 |  |
| No | 63 | 48.1 | 86 | 65.6 |  |
| Unsure | 38 | 29.0 | 28 | 21.4 |  |
| **Only need Anti D after birth if baby has a Rhd+ blood group** |  |  |  |  | .004 |
| Correct | 52 | 39.7 | 79 | 60.3 |  |
| Wrong | 41 | 57.7 | 30 | 42.3 |  |
| Unsure | 38 | 62.3 | 23 | 37.7 |  |
